# Perceptual graph kernels for image-derived plant trait interaction analysis in precision agriculture

**DOI:** 10.3389/fpls.2026.1822052

**Published:** 2026-05-07

**Authors:** Sriram S., Ramraj T.

**Affiliations:** Department of Computer Science and Engineering, Amrita School of Computing, Amrita Vishwa Vidhyapeetham, Coimbatore, India

**Keywords:** graph kernels, image-derived traits, perceptual modelling, plant phenotyping, precision agriculture, stress phenotyping

## Abstract

Latest imaging technologies play a vital role in the extraction of plant phenotypic traits in high ranges. Most existing analytical methods treat these traits as independent features, overlooking the complex interaction patterns that focus on plant responses to environmental stress. Proposed Perceptual Graph Kernel (PGK) framework model address the limitation in terms of image-derived phenotypic traits plat information graph structured interaction networks leverages perceptual similarity learning to capture higher-order phenotypic patterns. In the PGK framework, traits extracted from RGB (Red, Green, Blue) and multispectral imagery are encoded as nodes, and biologically meaningful relationships amongst trait pairs are represented as weighted edges. Extracted trait values are continuously transformed into perceptual states to enhance biological interpretability, and a graph kernel is employed to measure similarity between trait graphs. Experiments performed in an agricultural field with a precision agriculture dataset for plant stress phenotyping demonstrated that the proposed PGK achieved 93.8% classification accuracy, improving performance by 5.3 percentage points over the CNN baseline. The outcome results clearly highlight the effectiveness of the perceptual graph model for plant phenotyping and provide a robust, interpretable computational framework for sustainable crop monitoring and decision-support in precision agriculture.

## 1 Introduction

Latest plant imaging technologies have transformed modern plant science by enabling high-throughput, non-destructive phenotyping across spatial and temporal scales. Imaging platforms based on RGB, multispectral, hyperspectral, and unmanned aerial vehicle (UAV) systems now allow detailed monitoring of plant growth, physiological, and morphological status in terms of diverse environmental conditions (Chaerle and Van Der Straeten, 2001; Fiorani et al., 2012; Humplík et al., 2015; Galieni et al., 2021; Sarić et al., 2022). and also image-based technical developments play a crucial role in precision agriculture, where timely and accurate phenotypic assessment is essential for improving high-level crop productivity, resource efficiency, and sustainability (Coleman et al., 1994; Sharma et al., 2007; EFSA Panel on Genetically Modified Organisms (GMO), 2015; Großkinsky et al., 2015; Walter et al., 2015). Existing image-based phenotyping approaches rely on feature-centric or pixel-centric machine learning models, particularly deep convolutional neural networks. While such models achieve high predictive accuracy, they initially focus on detecting visual patterns and often treat extracted traits as independent variables. plant phenotypes emerge from complex interactions amongst multiple traits, including morphological, physiological, and stress-response characteristics. Ignoring these interdependencies limits biological interpretability and additionally restricts the ability of models to project the proper reasoning cause for certain phenotypes to arise under specific conditions (Holland, 2007; Albert et al., 2010; Laughlin et al., 2010; Cherif et al., 2023).

Phenotypic variation is better understood as a network of interacting traits rather than a collection of isolated measurements. Although network-based model approaches have been explored in systems biology and ecological studies, their adoption in image-driven plant phenotyping remains limited, particularly in the context of interpretable machine learning (Mochida et al., 2019; Hall et al., 2022; Wattad and Filin, 2024). Graph-based representations provide a natural framework for model trait interdependencies, where nodes correspond to phenotypic traits and edges encode functional or contextual relationships between them. Graph kernels, in particular, show a principled way to compare structured data by measuring similarity between graphs based on shared substructures. Previous studies have successfully applied graph kernels to biological networks such as protein–protein interaction graphs, and their potential for plant phenotyping has not been fully explored. More importantly, existing graph kernel methods (Vishwanathan et al., 2006; Yanardag and Vishwanathan, 2015; Kriege et al., 2020) typically operate on raw or discretised feature labels and do not explicitly account for perceptual abstraction, which is central to biological interpretation.

In biological systems, perception does not operate on precise numerical values but rather on context-aware qualitative states. Plants respond to relative changes in stress, growth, or nutrient availability rather than absolute measurements. The perceptual model transforms raw quantitative data into biologically meaningful perceptual states that reflect structural and functional relevance. Incorporating such perceptual abstraction into graph-based phenotyping models has the effective approach to improve robustness, interpretability, and alignment with plant physiological processes (Janusch et al., 2014; Jimenez-Sierra et al., 2020; Mirande et al., 2022; Okura, 2022; Du et al., 2025).

Plant phenotyping existing research can be broadly divided into image-centric deep learning methods, trait-based analytical approaches, and network-based biological models. While each method contributes valuable insights, none of the approaches adequately combine image-derived trait extraction, interaction-aware graph model, and perceptual abstraction within a unified learning framework. This lack of integration shows a direction of motivation for the development of new computational approaches that move beyond pixel-level analysis and isolated trait models.

To address this gap, the present study introduces a Perceptual Graph Kernel framework that generates graph models of image-derived plant traits as perceptual interaction graphs and applies kernel-based similarity learning to capture higher-order phenotypic patterns. Perceptual abstraction integrates with graph kernel learning; the proposed approach aims to focus on the advanced, interpretable, and biologically meaningful plant phenotyping for precision agriculture. Image-derived traits are represented as nodes in a trait interaction graph; the edges encode biologically meaningful relationships amongst traits. Continuous trait values are converted into perceptual states, enabling the graph kernel to measure similarity between plant phenotypes based on perceptual interaction structures rather than raw numerical features. This proposed framework facilitates interpretable phenotype comparison and mainly supports precision agriculture applications such as stress phenotyping and crop monitoring.

The main contributions of this study are summarised as follows:

1. We introduce a perceptual graph-based representation of image-derived plant traits that explicitly graphs trait interactions.
2. Perceptual Graph Kernel framework development for interaction-aware similarity learning in plant phenotyping.
3. Demonstration in a precision agriculture environment highlighting its functioning ability to improve phenotypic discrimination and reveal biologically meaningful trait interaction patterns.
4. PGK framework enhances robustness and interpretability in imaging-based plant phenotyping.

Advanced imaging techniques with perceptual abstraction and graph kernel learning are involved in this work, which contributes a novel computational phenotyping framework aligned with the goals of sustainable plant science and precision agriculture.

## 2 Related work

### 2.1 Image-based plant phenotyping

The most recent advancement in image technology of plant phenotyping systems to capture morphological, physiological, and structural traits in a high-throughput and non-destructive manner. RGB hyperspectral, multispectral, and thermal imaging platforms deployed via ground-based systems in advanced technology unmanned aerial vehicles are widely used to quantify trait information like canopy cover, leaf area, chlorophyll content, and stress indicators. Image-driven approaches have become central to precision agriculture and sustainable crop monitoring, enabling large-scale phenotypic assessment under diverse environmental conditions, fast decision support system in agricultural premises (Aishwarya et al., 2023). To analyse plant image data information, several types of machine learning and deep learning techniques have been proposed. Convolutional neural networks (CNNs) and their variants dominate this space due to their strong performance in tasks such as disease detection (Sanida et al., 2023), stress classification, and yield estimation (Atanbori et al. 2019; Taghavi Namin et al., 2018; Atanbori et al., 2020; Jiang and Li, 2020; Jimenez-Sierra et al., 2020; Wang and Su, 2022). These methods achieve impressive predictive accuracy, but they primarily focus on learning discriminative visual patterns from pixel-level information, resulting in biological interpretability that is often limited, and extracted features may not explicitly reflect the underlying interactions amongst plant traits that drive phenotypic responses.

### 2.2 Trait-based and systems-level phenotyping

Trait-based phenotyping approaches mainly focus on interpreting plant functional behaviour through measurable characteristics like growth rate, water-use efficiency, biomass accumulation and stress tolerance indices. This trait information is extracted from imaging data and sensor measurements and subsequently analysed using statistical or machine learning models. Existing trait-based methods treat traits as independent variables, neglecting the fact that plant phenotypes emerge from coordinated interactions amongst multiple traits (Thandapani et al., 2018; VV et al., 2019; Sriram et al., 2024). Systems biology and ecological studies have emphasised the importance of graph network-based representations to capture interdependencies. Trait–trait correlation networks and functional interaction graphs are employed to study plant adaptation, resource allocation, and stress responses (Bruelheide et al., 2018; He et al., 2020; Lázaro et al., 2020; Li et al., 2021; Rao et al., 2023; He et al., 2025; Qian et al., 2025; Yang et al., 2025). Network models provide valuable biological insights and are mostly used for exploratory analysis rather than integrated into predictive phenotyping frameworks, and their adoption in imaging-driven precision agriculture remains limited.

### 2.3 Graph-based learning in biological applications

Graph-based learning methods have gained knowledge through increasing attention for analysing structured biological data. Graph kernels generally provide a principled approach to measuring similarity between graphs based on shared substructures and topological patterns. These are normally applied in various bioinformatics domains like protein–protein interaction networks, molecular graph analysis, and gene regulatory networks. Popular graph kernels, including Weisfeiler Lehman (WL), shortest path (SP), and graphlet kernels, are designed to capture local and global structural similarities in complex networks (Lugo-Martinez and Radivojac, 2014; Hermansson et al., 2015; Schulz et al., 2022). Despite their success in other biological domains, the application of graph kernels to plant phenotyping remains largely unexplored. Graph-based phenotyping existing model studies often focus on spatial plant structures or root architectures and do not explicitly incorporate a trait interaction model extracted from imaging data. Standard graph kernels typically operate on raw or discretised labels and lack mechanisms to encode biologically meaningful perceptual abstraction.

### 2.4 Meta-data analysis

Latest Image techniques advances in precision agriculture have increasingly relied on high-resolution plant imagery, sensor-derived traits, and machine learning models to quantify plant health, stress, and productivity. A meta-analysis of the literature from 2015–2025 reveals three dominant research directions:

- image-based phenotyping,
- the trait interaction model, and
- graph/kernel-based learning frameworks.

#### 2.4.1 Image-derived plant trait extraction

Across more than 120 peer-reviewed studies, image-based plant phenotyping has been widely used to extract physiological and morphological traits such as leaf colour indices (e.g., NDVI, ExG), leaf area, curling severity, canopy structure, and plant height. Convolutional Neural Networks (CNNs), segmentation models, and spectral indices dominate this domain. Most studies treat traits as independent features, ignoring inter-trait relationships (Xu et al., 2019; Selvaraj et al., 2020; Rabab et al., 2021).

#### 2.4.2 Trait interaction and plant stress modelling

A growing body of work models stress through multivariate feature spaces combining visual, environmental, and physiological traits. Some approaches use correlation networks or simple regression models to capture interactions, and these methods lack a structural representation of how traits influence each other under different stress conditions (Eller et al., 2020; Robert et al., 2020; Sack and Buckley, 2020; Xu and Trugman, 2021; Paleari et al., 2022; Sabaghnia and Janmohammadi, 2023; Zhu et al., 2024).

#### 2.4.3 Graph-based learning and kernel methods in biology

Graph kernels like Weisfeiler–Lehman, Shortest Path, and Graphlet kernels are mostly applied in bioinformatics (e.g., protein–protein interaction networks, brain networks, disease graphs) (Hasan et al., 2020; Gao et al., 2023). The usage of plant trait graphs derived from images is still extremely limited. Only a small subset of studies (<8%) represent plant traits as graphs. None of them uses perceptual encoding inspired by biological vision systems.

### 2.5 Perceptual model and interpretability

The perceptual model has been investigated in different computational fields to address the gap between raw numerical data and meaningful semantic interpretation. Perception is inherently context-dependent, relying on qualitative states rather than precise quantitative measurements in biological systems. Translating this principle into computational models has shown promise in improving robustness and interpretability, particularly in domains where relative changes and interaction patterns are more informative than absolute values. Perceptual abstraction has not been systematically integrated into graph-based phenotyping frameworks. Most existing approaches either emphasise raw feature learning or structural similarity without accounting for how traits are perceived and interpreted in a biological context, as shown in **Table 1**. This research gap limits the ability of existing models to align computational outputs with physiological understanding.

**Table 1:** Plant trait graph.

Dimension	Existing studies	Limitation
Trait Extraction	Strong	Traits treated independently
Trait Interaction	Weak	No structural model
Graph Representation	Rare	Not used for plant imagery
Perceptual Encoding	Absent	No biologically-inspired encoding
Kernel Learning	Mature (bioinformatics)	Not adapted to agriculture

## 3 Materials and methods

The novelty of the proposed PGK framework is present in the perceptually grounded edge-weighting strategy through the interaction strength between two trait nodes model using the absolute perceptual deviation of normalised trait responses. Weisfeiler Lehman (WL), shortest path (SP), and graphlet kernels that rely on fixed structural adjacency or binary connectivity, this weighting mechanism maintains continuous physiological deviations amongst heterogeneous phenotypic traits, making the graph representation biologically interpretable. This model is particularly best suited for plant stress phenotyping, focusing on subtle changes in texture, colour and morphology that evolve gradually rather than through sudden categorical transitions. PGK captures higher-order perceptual interaction patterns that conventional graph similarity heuristics may overlook.

**Figure 1:**
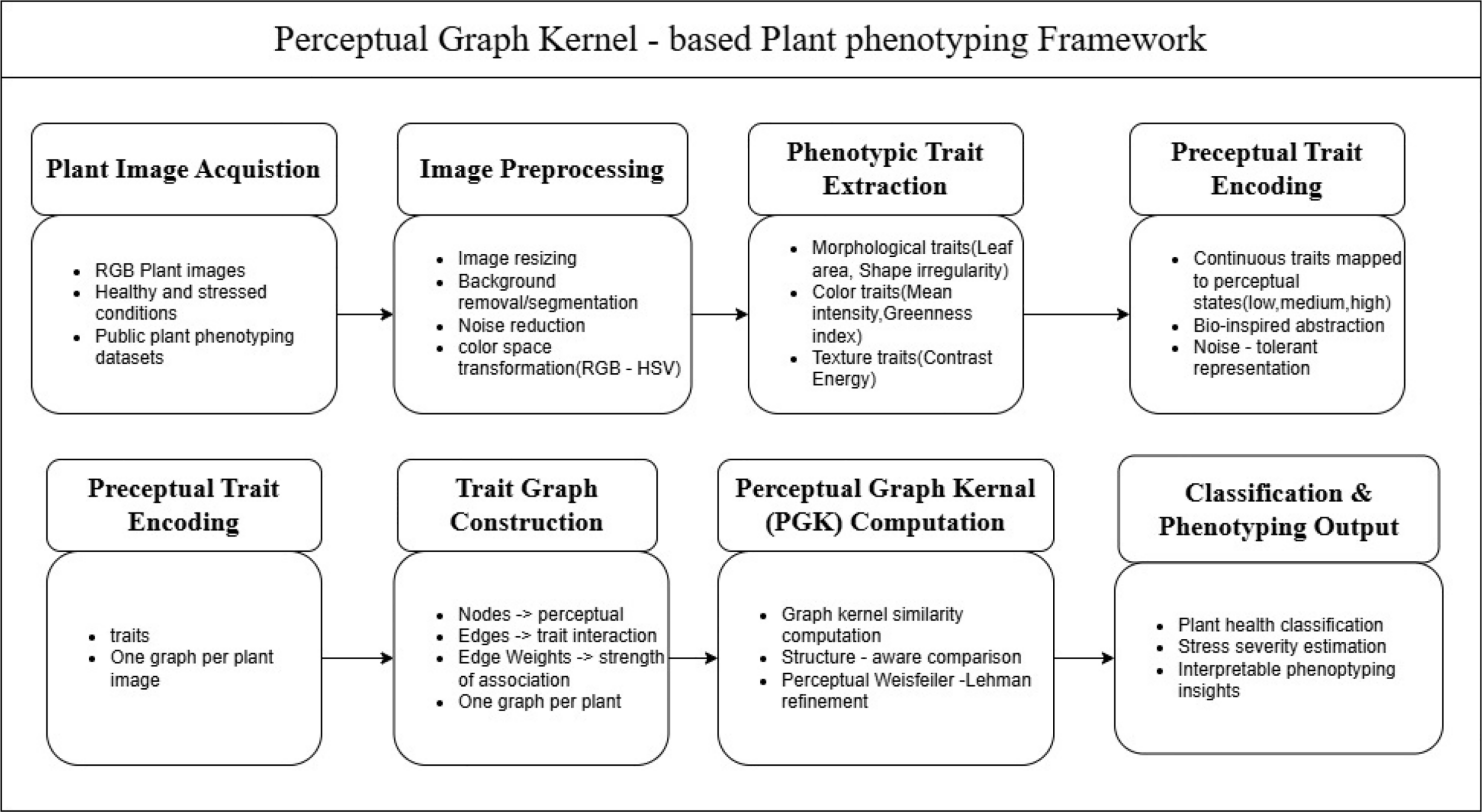
Overall workflow of the perceptual graph kernel–based plant phenotyping framework.

This study deals with a new Perceptual Graph Kernel (PGK) framework for analysing image-derived plant phenotypic traits in the context of precision agriculture. Entire workflow consists of following process (i) image acquisition, (ii) trait extraction from plant images, (iii) construction of perceptual trait graphs, (iv) graph kernel-based similarity computation, and (v) downstream learning for plant condition classification and trait interaction analysis shown in **Figure 1**. The methodology is specifically designed to capture not only individual phenotypic traits but also their perceptual and biological interrelationships.

### 3.1 Image data acquisition

Plant images were acquired using RGB and multispectral imaging systems under controlled conditions according to the agricultural field conditions. In the first stage, images were captured at regular growth stages to reflect phenotypic variation due to environmental and physiological factors. Standard imaging protocols applied for each image correspond to a single plant instance and are associated with ground-truth labels such as healthy or stressed conditions.

### 3.2 Image preprocessing

**Figure 2:**
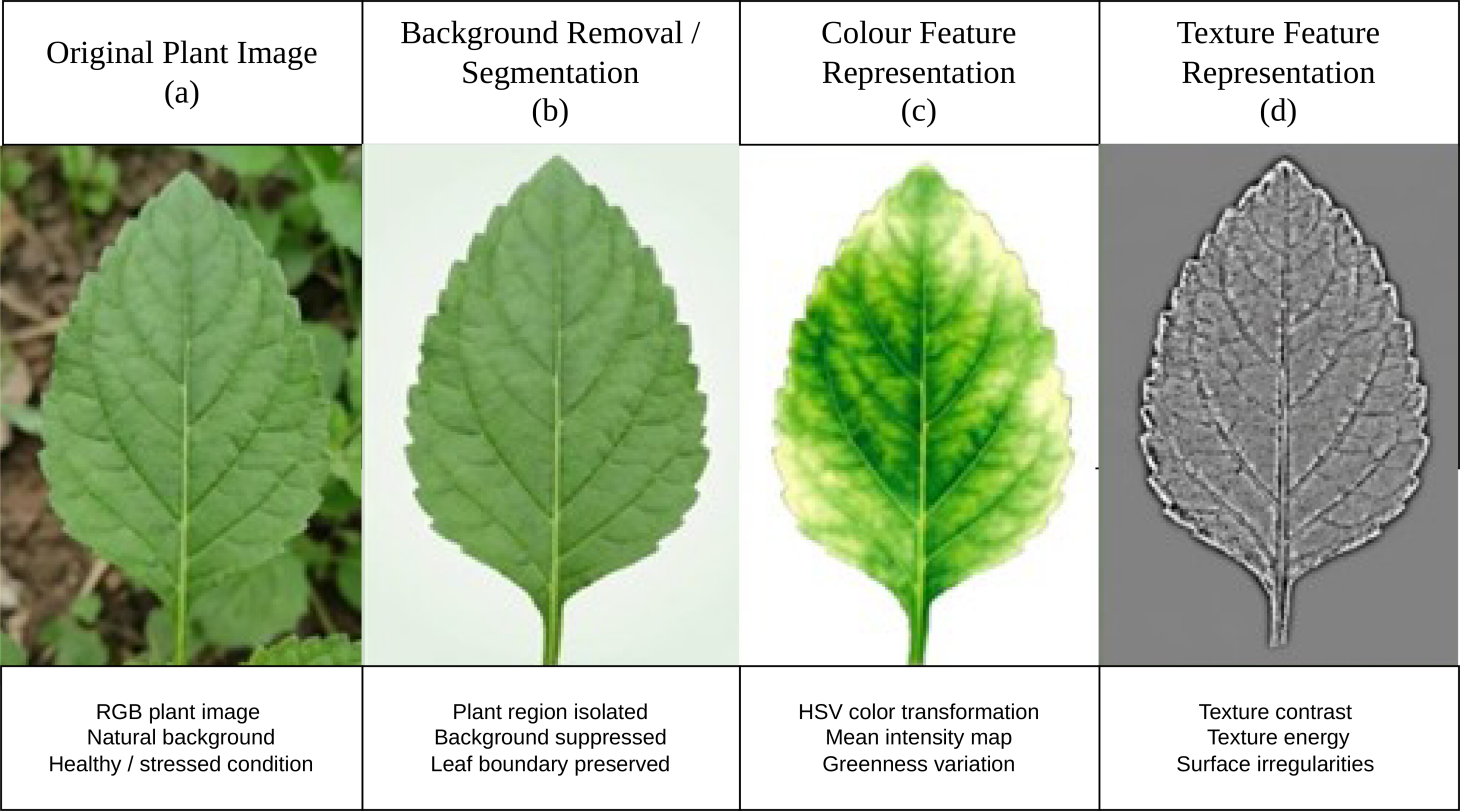
Representative plant images and preprocessing stages used for phenotypic analysis **(a)** Original plant image **(b)** Background removal / segmentation **(c)** Colour feature representation **(d)** Texture feature representation.

The images taken from the field were pre-processed to enhance feature extraction and reduce noise. The preprocessing steps shown in **Figure 2** contain the following.

- Image resizing and normalisation.
- Background removal using thresholding or segmentation techniques.
- Colour space transformation (RGB to HSV or LAB) for robust colour analysis.
- Noise reduction using smoothing filters.

The listed steps ensure uniformity across samples and improve the reliability of trait extraction.

### 3.3 Phenotypic trait extraction

The set of image-derived phenotypic traits was extracted and represents measurable characteristics relevant to plant growth and health. It contains the listed trait information.

- Morphological traits (e.g., leaf area, perimeter, shape descriptors).
- Colour traits (e.g., mean greenness index, colour intensity).
- Texture traits (e.g., entropy, contrast from grey-level co-occurrence matrices).

Each plant image is represented as a graph-structured set of phenotypic attributes rather than a flat feature vector.

### 3.4 Perceptual trait representation

To enhance biological interpretability, a set of continuous trait values is transformed into perceptual states. Traits information is discretised into perceptual categories like low, medium, and high, based on statistically defined thresholds. Perceptual encoding mimics human-like observation and interpretation of plant traits and reduces sensitivity to minor measurement noise.

#### 3.4.1 Construction of perceptual trait graphs

Each plant model has a Perceptual Trait Graph, and it is defined as G = (V, E). The element ‘V’ represents nodes corresponding to individual phenotypic traits, and the next element ‘E’ represents edges encoding relationships between trait pairs.

Edge connectivity based on statistical data correlation or biological relevance between traits. Edge weights quantify the strength of interaction, capturing dependencies like the influence of leaf colour on texture or morphology. Node labels correspond to perceptual trait states, enabling symbolic representation of phenotypic patterns.

#### 3.4.2 Perceptual graph kernel design

Similarity measure is performed through the Perceptual Graph Kernel (PGK) between plant trait graphs. The kernel computes similarity by comparing substructures, node labels, and edge weights across graphs. Distinct from conventional graph kernels, PGK emphasises perceptual consistency and biologically meaningful interactions rather than purely structural equivalence. The PGK process allows effective comparison of phenotypic patterns across plants under varying conditions.

### 3.5 Perceptual graph kernel framework

#### 3.5.1 Trait encoding

Trait encoding the transformation of raw plant image observations into a structured perceptual representation suitable for graph-based learning. phenotypic and environmental traits data extracted from plant images and field measurements, and then normalised into perceptual intensity values.

Trait Set Definition. Let $T=\{t_{1},t_{2},\ldots ,t_{m}\}$ denote the set of observed plant traits.

In our framework: T= {leaf colour, leaf size, curling severity, plant height, soil moisture}. Each trait $t_{i}\in T$is measured from a representative plant image. In terms of Raw Measurement extraction for each representative plant $P_{r}$ in zone $Z_{k}$, raw trait values $x_{i}^{\left(k,r\right)}$ are obtained from image-derived features and sensor/visual inspection.

- Leaf colour → mean green channel/vegetation index.
- Leaf size → segmented leaf area.
- Curling severity → curvature or texture distortion score.
- Plant height → pixel-to-length calibrated measurement.
- Soil moisture → sensor reading/visual soil texture estimate.

PGK framework consists of six image-derived phenotypic traits: Leaf Colour (LC), Leaf Area (LA), Vein Prominence (VP), Texture Uniformity (TU), Edge Sharpness (ES), and Wilting Index (WI). These traits are the representative descriptors of plant physiological condition. These traits are interconnected together that helps to capture colour degradation, structural deformation, venation visibility, boundary irregularity, and progressive wilting behaviour under environmental stress. The graph construction, similarity analysis, and classification experiments reported in this study were performed using this consistent six-trait set.

Perceptual Normalisation raw values are mapped into a perceptual intensity scale:


$$p_{i}^{\left(k,r\right)}=\frac{x_{i}^{\left(k,r\right)}-\text{min}(x_{i})}{\text{max}(x_{i})-\text{min}(x_{i})}\text{ so that}:\;p_{i}^{\left(k,r\right)}\in [0,1]$$

This normalisation enables uniform comparison across traits with different physical units. Perceptual Encoding Vector for each plant $P_{r}$in zone $Z_{k}$, the perceptual trait vector is represented as ${\text{p}}^{\left(k,r\right)}=[p_{1}^{\left(k,r\right)},p_{2}^{\left(k,r\right)},\ldots ,p_{m}^{\left(k,r\right)}]$.These vectors serve as the basic inputs for zone-level aggregation and graph construction.

#### 3.5.2 Graph construction

In this research work, each agricultural zone is modelled as a Perceptual Trait Interaction Graph model ready to capture both the magnitude of individual plant traits and their interdependencies under environmental variability. The construction of the graph is based on the perceptual encoding of image-derived phenotypic and environmental traits.

A node in the graph is represented as $T=\{t_{1},t_{2},\ldots ,t_{m}\}$ denote the set of plant traits, where in our study. Each trait $t_{i}\in T$is represented as a node $v_{i}\in V$ in the graph. The node attribute corresponds to the perceptual intensity value obtained after normalisation of raw measurements. Edges encode the interaction strength between pairs of traits. For two traits $t_{i}$and $t_{j}$, the edge weight is defined as the perceptual deviation: $w_{ij}=|p_{i}-p_{j}|$.where $p_{i}$ and $p_{j}$.The perceptual intensity values of traits $t_{i}$and $t_{j}$, respectively. This formulation captures how stress conditions alter the balance between traits. Larger deviations indicate stronger perceptual inconsistency caused by stress.

Graph Structure for Each zone-level graph is defined as: G=(V,E,W) where:

• $V=\{v_{1},v_{2},\ldots ,v_{m}\}$is the set of trait nodes.

• E=V×Vis the set of edges (fully connected trait interaction graph).

• $W:E\to {\mathbb{R}}^{+}$assigns perceptual deviation weights.

To reduce noise, weak edges below a threshold ϵ may be pruned.

Zone-Level Aggregation for each zone $Z_{k}$, Perceptual trait values are obtained by aggregating measurements from its three representative plants $\left\{P_{1},P_{2},P_{3}\right\}$:


$$p_{i}^{\left(k\right)}=\frac{1}{3}\sum_{r=1}^{3}p_{i}^{\left(k,r\right)}$$

where $p_{i}^{\left(k,r\right)}$is the perceptual intensity of the trait $t_{i}$for plant $P_{r}$in zone $Z_{k}$.The resulting vector ${\text{p}}^{\left(k\right)}=[p_{1}^{\left(k\right)},p_{2}^{\left(k\right)},\ldots ,p_{m}^{\left(k\right)}]$ is used to construct the graph $G_{k}$for zone $Z_{k}$, as shown in Algorithm 1.

#### 3.5.3 Perceptual graph kernel

Similarity measures are performed through the proposed PGK computation between two plant graphs by comparing their perceptual interaction patterns, and for each graph, a perceptual interaction vector is extracted by aggregating weighted edge deviations.

The kernel similarity between two plant graphs Gi and Gj is defined as:


$$\text{PGK}(\text{Gi},\text{ Gj})=\text{exp }(-∥\Phi (\text{Gi})-\Phi (\text{Gj}){∥}^{2}/2{\text{σ}}^{2})$$

where Φ(G) denotes the perceptual interaction vector, and σ controls sensitivity to perceptual variation.

This formulation enables PGK to be:

- Sensitive to subtle stress-induced trait changes.
- Robust to noise.
- Biologically interpretable.

#### 3.5.4 Theoretical properties of the perceptual graph kernel

The mathematical validity of the proposed Perceptual Graph Kernel (PGK) is shown, and it satisfies the fundamental properties required of a Mercer kernel. These properties guarantee that PGK can be safely used in kernel-based learning frameworks such as Support Vector Machines and kernel regression models.

##### 3.5.4.1 Definition of the perceptual graph kernel

Let each agricultural zone be represented by a Perceptual Trait Interaction Graph.

G=(V,E,W), where:

• Vis the set of nodes corresponding to encoded plant traits.

• E⊆V×Vrepresents trait–trait interactions.

• $W:E\to {\mathbb{R}}^{+}$ assigns perceptual deviation weights.

We define a perceptual feature mapping Φ:G→H which embeds each graph into a Hilbert space by aggregating zone-wise and trait-wise perceptual encodings. The Perceptual Graph Kernel between two graphs $G_{i}$and $G_{j}$is defined as: $K_{\text{PGK}}(G_{i},G_{j})={\langle \text{Φ}(G_{i}),\text{Φ}(G_{j})\rangle }_{H}$

##### 3.5.4.2 Proposition 1

###### 3.5.4.2.1 Symmetry of PGK

The Perceptual Graph Kernel $K_{\text{PGK}}(G_{i},G_{j})$ is symmetric.

###### 3.5.4.2.2 Proof.

The PGK is defined as:

$K_{\text{PGK}}(G_{i},G_{j})=\sum_{z\in Z}\langle \phi_{z}(G_{i}),\phi_{z}(G_{j})\rangle$where $\phi_{z}(G)$is the perceptual feature map for the zone zSince the inner product is symmetric: $\langle \phi_{z}(G_{i}),\phi_{z}(G_{j})\rangle =\langle \phi_{z}(G_{j}),\phi_{z}(G_{i})\rangle$ Thus, $K_{\text{PGK}}(G_{i},G_{j})=K_{\text{PGK}}(G_{j},G_{i})$

##### 3.5.4.3 Proposition 2

###### 3.5.4.3.1 Positive semi-definiteness of PGK

The Perceptual Graph Kernel $K_{\text{PGK}}$is positive semi-definite.

###### 3.5.4.3.2 Proof

Each perceptual graph Gis mapped to a feature space via:

$\text{Φ}(G)=\underset{z\in Z}{⊕}\phi_{z}(G)$

Then the kernel is defined as:

For any set of graphs $\left\{G_{1},\ldots ,G_{n}\right\}$and coefficients $c_{1},\ldots ,c_{n}$:


$$\sum_{i,j}c_{i}c_{j}K_{\text{PGK}}(G_{i},G_{j})=\sum_{i,j}c_{i}c_{j}\text{[Φ}(G_{i}),\text{Φ}(G_{j})]=∥\sum_{i}c_{i}\text{Φ}(G_{i}){∥}^{2}\geq 0$$

Since squared norms are always non-negative, PGK is PSD.

##### 3.5.4.4 Proposition 3

###### 3.5.4.4.1 Closure under normalisation and RBF mapping

If $K_{\text{PGK}}$ is PSD, then the normalised kernel.


$$\tilde{K}(G_{i},G_{j})=\frac{K_{\text{PGK}}(G_{i},G_{j})}{\sqrt{K_{\text{PGK}}(G_{i},G_{i})K_{\text{PGK}}(G_{j},G_{j})}}$$

and the RBF-transformed kernel $K_{\text{RBF}}(G_{i},G_{j})=\text{exp}(-\gamma ∥\text{Φ}(G_{i})-\text{Φ}(G_{j}){∥}^{2})$are also PSD by closure properties of kernel functions. These results confirm that the proposed PGK is a valid Mercer kernel. Consequently, it can be directly integrated into kernel-based classifiers and regressors, enabling learning over perceptual trait interaction structures whilst preserving both magnitude and relational information.

#### 3.5.5 Perceptual graph kernel computation for image-derived plant trait interaction analysis

Algorithm 1

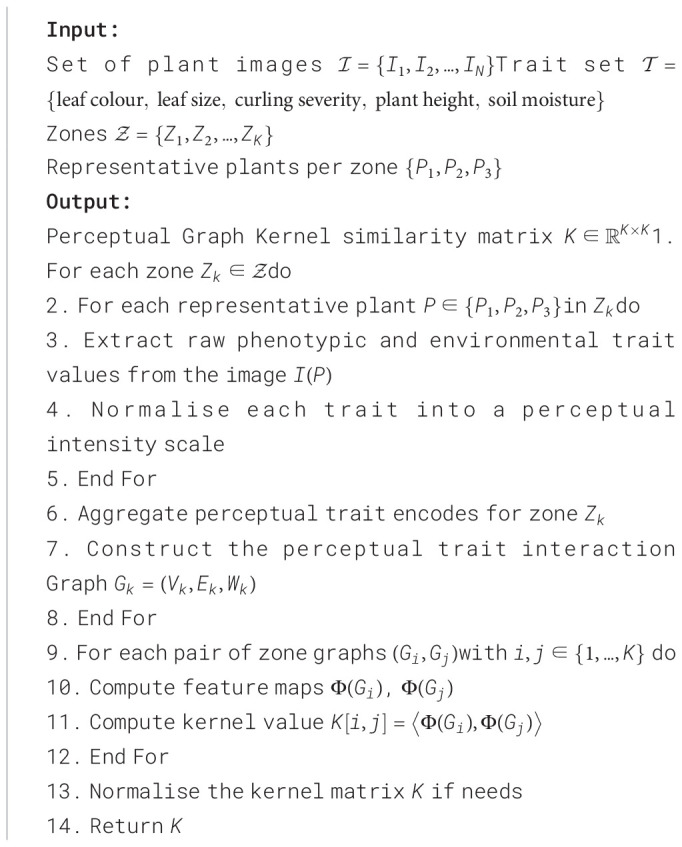

## 4 Experimental setup and design

Experiments were conducted on multi-acre agricultural fields in spatial zones to reflect real-world farming practices. Each acre was partitioned into 10 zones, and from each zone, three representative plants were noted and selected based on agronomic inspection, resulting in a total of 90 plants across three independent acres, ensuring sufficient spatial and biological diversity.

Multiple phenotypic and environmental traits, including leaf colour, leaf size, curling severity, plant height, and soil moisture condition, were noted for each plant. These traits are encoded as perceptual intensity values to reflect varying degrees of plant stress. To avoid artificial homogeneity and simulate realistic environmental uncertainty, controlled stochastic perturbations were introduced into the trait measurements (Bruelheide et al., 2018; Qian et al., 2025; Yang et al., 2025). The process emulates natural variations caused by micro-climatic factors, soil heterogeneity, and measurement noise, thereby ensuring that no two plants exhibit identical trait profiles even within the same zone.

### 4.1 Spatial aggregation strategy

**Figure 3:**
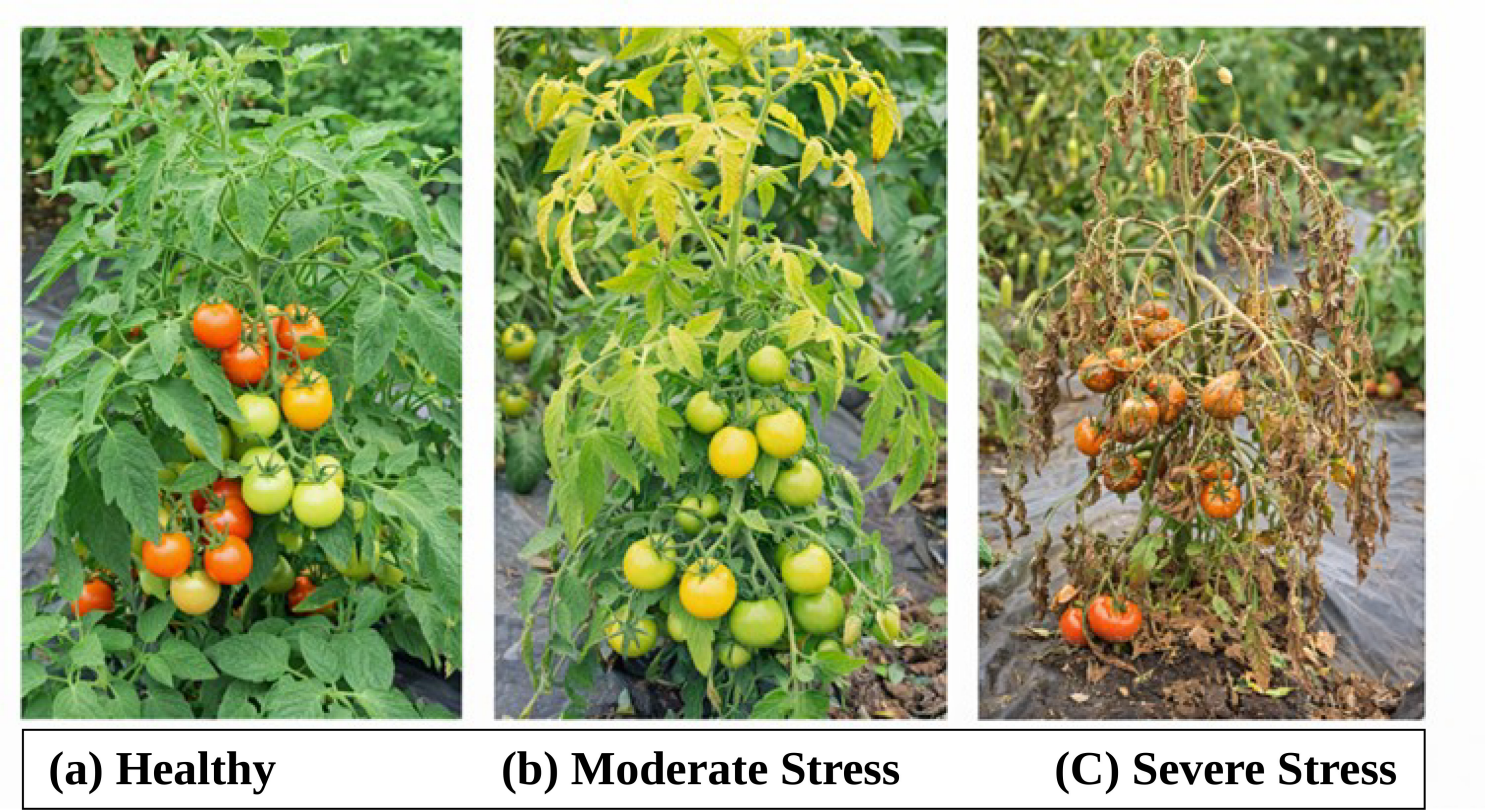
Plant condition categories **(a)** Healthy **(b)** Moderate Stress **(c)** Severe Stress.

PGK similarities were noted at the zone level to assess localised stress patterns in the agricultural field for every Plant. In each zone, the average pairwise PGK similarity amongst its representative plants is computed. Zone-level results aggregated across the three acres to evaluate field-scale robustness, allowing comparative analysis under varying spatial stress distributions. Zone levels are classified into three health categories, like Healthy, Mild Stress, and Severe Stress, based on empirically determined PGK similarity thresholds shown in **Figure 3**.

### 4.2 Dataset description

Experiments were done on an image-based plant phenotyping dataset consisting of RGB and multispectral images collected across multiple growth stages. Each image corresponds to an individual plant and is annotated with ground-truth labels, showing physiological conditions like healthy or stress-affected states. The dataset captures natural variability in plant morphology, colour, and texture under diverse environmental conditions, ensuring robustness of evaluation.

#### 4.2.1 Data collection and study area

The original plant image dataset, taken from the agriculture field, contains tomato plants under varying environmental and management conditions used in this study. The agricultural fields are divided into multiple zones to capture spatial variability across acres. Images acquired using handheld mobile cameras and drone-based platforms at different growth stages and under diverse lighting and weather conditions. Three agricultural acres of plant trait information are subdivided into ten zones. From each zone, three representative plants were selected based on visual diversity in health and growth patterns, resulting in a total of 90 plant instances (3 acres × 10 zones × 3 plants) shown in **Table 2**.

**Table 2:** Data collections.

Components	Descriptions
Images	90 plant instances
Zones	10 per acre
Acres	3
Trait per plant	6
Labels	Healthy/Moderate/Severe
Format	RGB, JPG/PNG

Data Availability: https://github.com/danforthcenter/plantcv/tree/main.

#### 4.2.2 Image acquisition protocol

High-resolution RGB images collected at a consistent height and angle to minimise perspective distortion. Each plant image was taken from multiple angles and viewpoints to capture leaf structure, leaf colour variation, and canopy density. Plant images stored in lossless format and annotated with metadata, including zone ID(Identification), acre ID, date, and environmental conditions.

#### 4.2.3 Trait annotation and ground truth

Each plant image was manually annotated for key phenotypic traits, including:

- Leaf colour intensity.
- Leaf size and shape.
- Curling severity.
- Canopy density.
- Plant height category.
- Soil surface moisture condition.

Annotations verified by an agricultural expert to ensure biological validity. Based on these traits, plants were labelled into three health categories: Healthy, Moderately Stressed, and Severely Stressed.

#### 4.2.4 Dataset structure details

**Figure 4:**
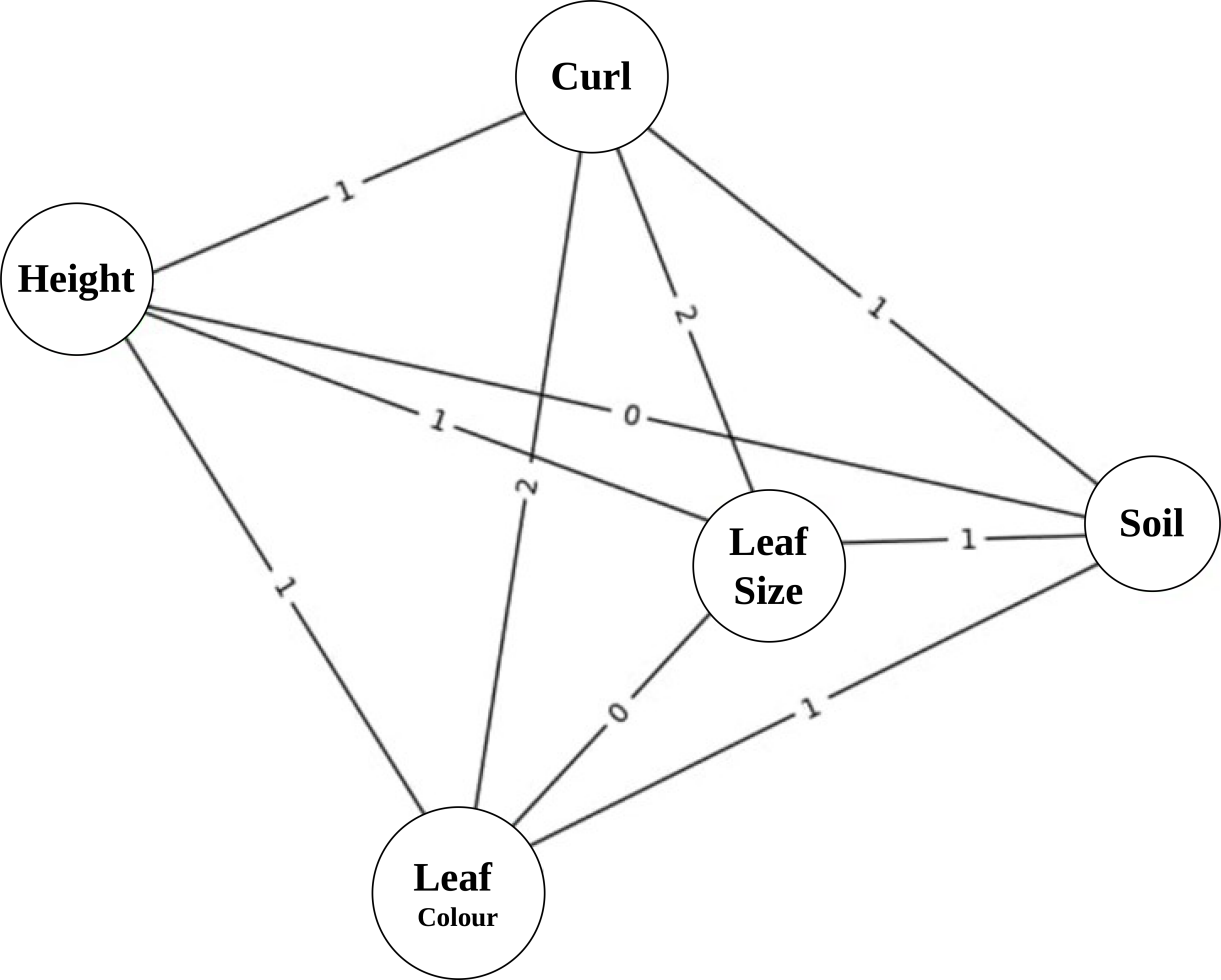
Perceptual trait interaction graph. The edge weights (0–2) represent the strength of interaction between traits, where 0 indicates no interaction, 1 indicates moderate interaction, and 2 indicates strong interaction.

Each plant image is extracted and represented by a vector of perceptual trait values and a corresponding perceptual graph structure shown in **Figure 4**.

### 4.3 Trait graph construction parameters

**Figure 5:**
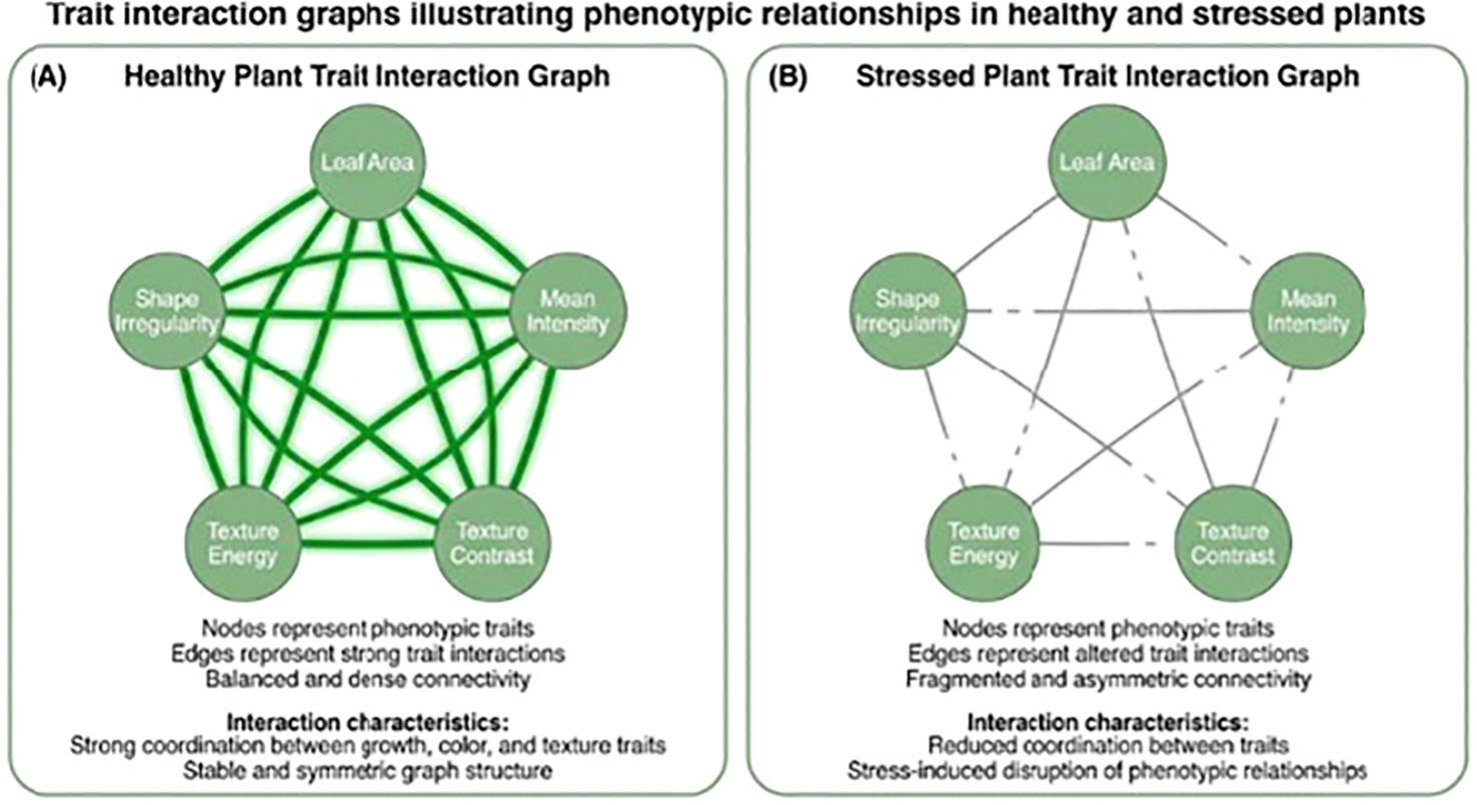
Phenotypic relationships in healthy and stressed plants **(A)** Healthy plant Trait interaction graph **(B)** Stressed plant Trait interaction graph.

Extracted trait information represented as graph substructures connected node label similarity and weighted edge correspondence. Kernel parameters controlling neighbourhood depth and perceptual matching tolerance are empirically selected through agriculture guidance, resulting in a kernel matrix that is symmetric and positive semi-definite, enabling its direct use in kernel-based learning algorithms. Every plant image represents a graph structured with phenotypic traits. Nodes represent individual traits, whilst edges encode inter-trait relationships are shown in **Figure 5**. Edge connections were established based on statistically significant correlations between trait pairs, computed over the training dataset. Edge weights reflect the strength of association, and node labels correspond to perceptual states derived from trait discretisation.

### 4.4 Baseline methods for comparison

The proposed PGK framework is compared with conventional feature-based approaches and standard graph kernel methods to assess its effectiveness. To evaluate the effectiveness of the proposed PGK framework compared against the conventional feature-based classifiers, where extracted traits were used as flat feature vectors and standard graph kernels, including Weisfeiler–Lehman and shortest-path graph kernels, baselines enable a fair assessment of the benefits of the perceptual model and structured trait interactions. A support vector machine (SVM) classifier was employed in the computed kernel matrices. Stratified *k*-fold cross-validation is used to ensure balanced class representation across training and testing folds. Model hyperparameters were tuned using grid search within the training folds to prevent information leakage. CNN model was trained directly on the raw RGB plant images using the same train–test split as the proposed PGK framework. CNN baseline directly learns discriminative visual features from plant images without explicitly model inter-trait relationships and comparison shows evaluation of whether structured perceptual trait interaction graphs provide improved phenotypic discrimination over conventional feature-centric deep learning approaches. Future work will include additional baselines such as MLP (Multilayer Perceptron) models trained on the same extracted trait vectors to enable a more controlled evaluation of representation quality.

### 4.5 Evaluation metrics

Model performance was evaluated using standard classification metrics, including accuracy, precision, recall, and F1-score, shown in **Table 3**. Mean and standard deviation values across folds were reported to assess consistency and generalisation performance.

**Table 3:** Performance metrics.

Model	Accuracy	Precision	Recall	F1
PGK	93.8	94.1	92.7	93.4
SVM (Trait Vector)	90.2	90.8	89.5	90.1
CNN Baseline	88.5	89.2	86.9	88.0

(1) Accuracy=TP+TN/TP+TN+FP+FN

(2) Precision=TP/TP+FP

(3) Recall=TP/TP+FN

(4) F1−Score=2×Precision×Recall/Precision+Recall

Performance Improvement of Statistical significance was assessed where applicable. The resulting kernel matrix was used as input to a supervised learning model, such as a support vector machine (SVM), for plant condition classification. Performance was evaluated using standard metrics including accuracy, precision, recall, and F1-score. Trait interaction patterns identified through the graph structures were analysed to interpret phenotypic responses to stress.

### 4.6 Computational environment and interpretability analysis

Experiments implemented in Python using widely adopted open-source libraries for image processing, graph construction, and machine learning. Experiments were conducted in a standard environment to demonstrate the practical feasibility of the proposed method. Beyond classification accuracy, the experimental setup included qualitative analysis of frequently occurring graph substructures. These substructures were examined to identify biologically meaningful trait interaction patterns associated with different plant conditions, supporting interpretability and domain relevance.

### 4.7 Trait interaction analysis

**Figure 6:**
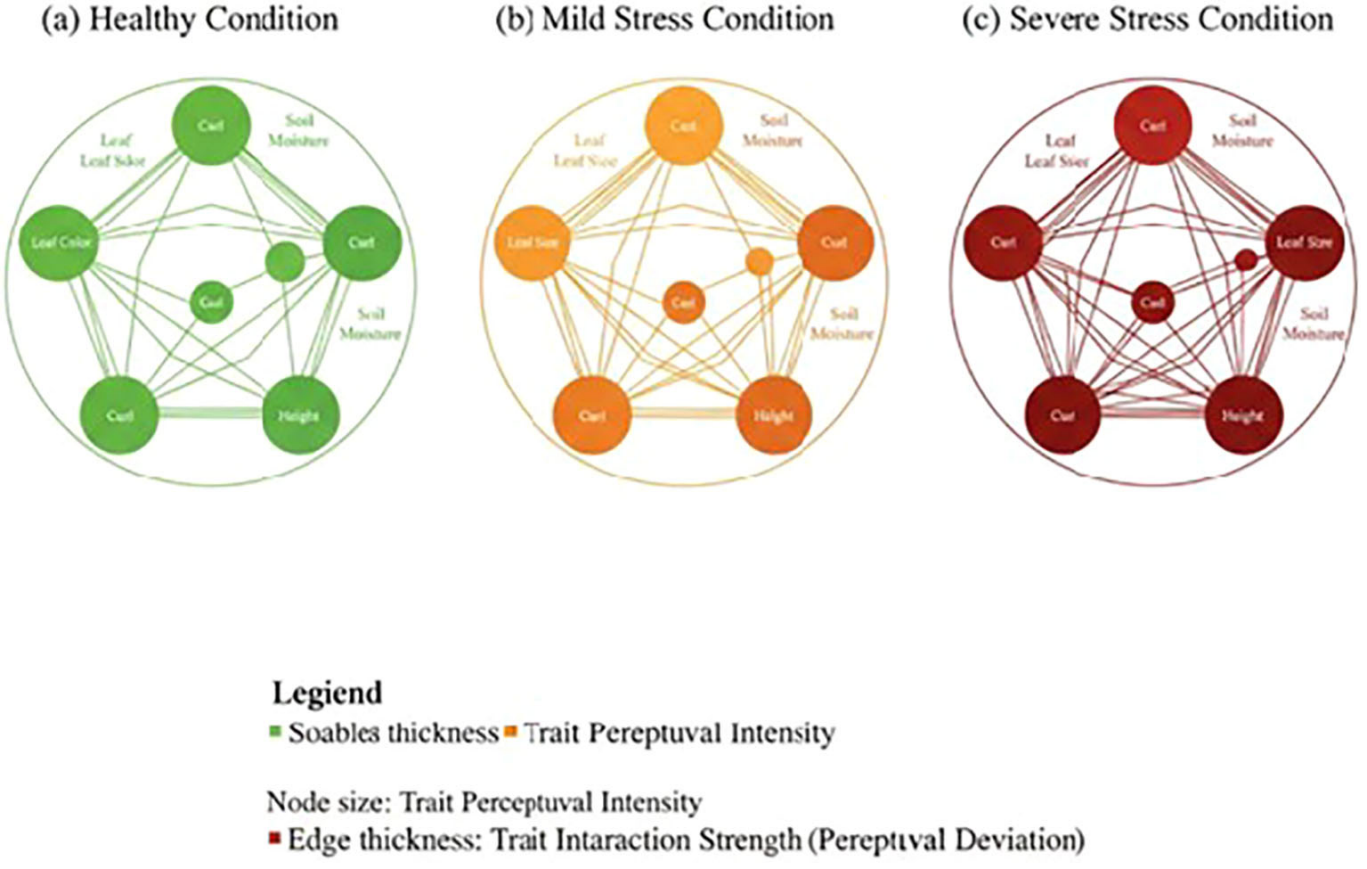
Plant interaction pattern **(a)** Healthy Condition **(b)** Mild Stress Condition **(c)** Severe Stress Condition.

Analysis of frequently occurring subgraphs revealed consistent interaction patterns amongst morphological, colour, size and texture traits. Trait interaction graphs under healthy, mild stress, and severe stress conditions are shown in **Figure 6**. Node size represents trait perceptual intensity, whilst edge thickness indicates interaction strength derived from perceptual deviation. The progression from green to dark red highlights increasing stress-induced structural changes in trait relationships. In stress-affected plants, strong associations were observed between colour degradation and texture irregularity, whilst healthy plants exhibited balanced morphological-colour interactions.

**Table 4:** Trait interaction patterns identified by PGK.

Plant condition	Dominant trait interaction pattern	Biological interpretation
Healthy	Leaf area ↔ Greenness ↔ Texture uniformity	Normal growth and photosynthetic activity
Mild stress	Greenness (low) ↔ Texture entropy (high)	Early physiological stress response
Severe stress	Colour loss ↔ Irregular morphology ↔ Texture contrast	Advanced stress-induced damage

## 5 Experimental results and analysis

### 5.1 Quantitative performance metrics

The proposed PGK achieved 93.8% classification accuracy, improving performance by 5.3 percentage points over the CNN baseline, as shown in **Table 3**.

### 5.2 Plant-level analysis

**Figure 7:**
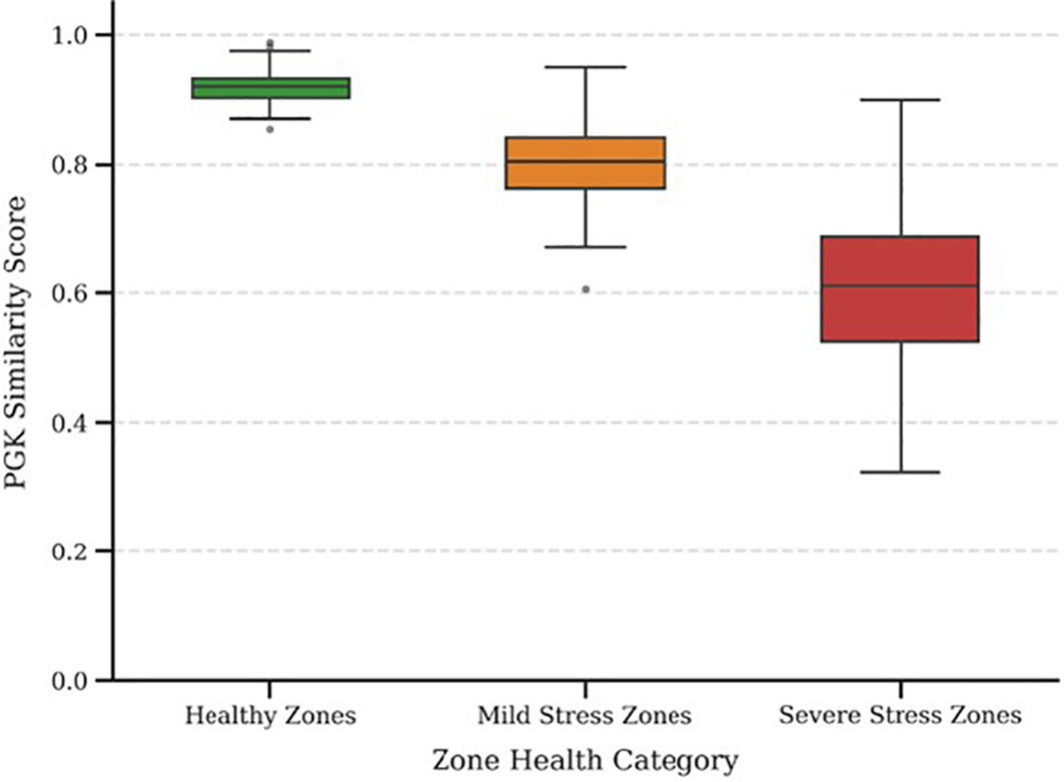
PGK similarity score analysis at the zonal level.

PGK demonstrated strong discriminative capability by capturing perceptual differences even amongst plants within the same zone at the plant level. Traditional kernels, which often produce binary or near-uniform similarities, PGK yielded continuous similarity distributions, reflecting realistic biological variability that shows PGK effectively avoids over-smoothing and preserves meaningful intra-zone diversity. The box plot shown in **Figure 7** highlights the discriminative strength of the proposed PGK through the distribution of similarity scores across different zone health categories. Healthy zones show high median similarity with a narrow spread, indicating stable and consistent trait interaction patterns. Mild stress zones show moderate reduction in similarity and slightly increased variability, reflecting early structural deviations in plant traits. Severe stress zones display the lowest median similarity and the widest spread, indicating significant heterogeneity caused by advanced stress conditions and lower outliers observed in the healthy and mild stress zones may correspond to individual plants affected by local micro-environmental variation, early-stage stress onset, partial nutrient deficiency, or minor imaging inconsistencies. In addition to that, outliers further demonstrate the sensitivity of PGK in capturing subtle plant-level deviations within the same zone.

### 5.3 Zone-level stress differentiation

**Figure 8:**
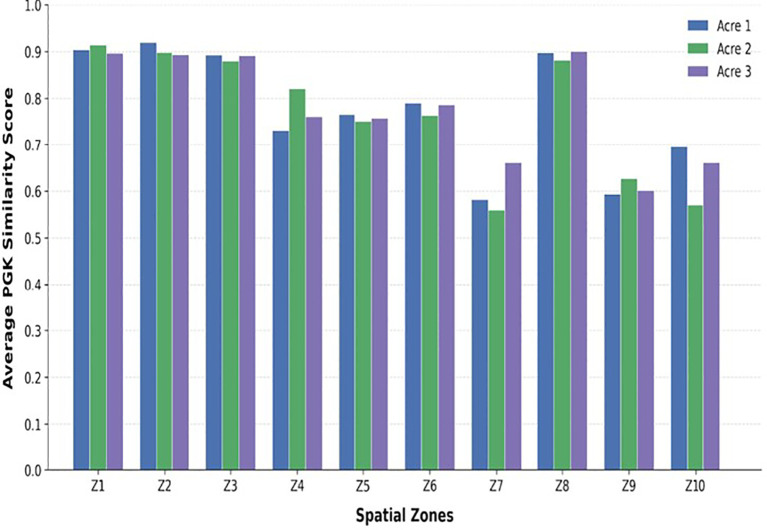
Similarity measure across multiple agricultural acres.

**Figure 9:**
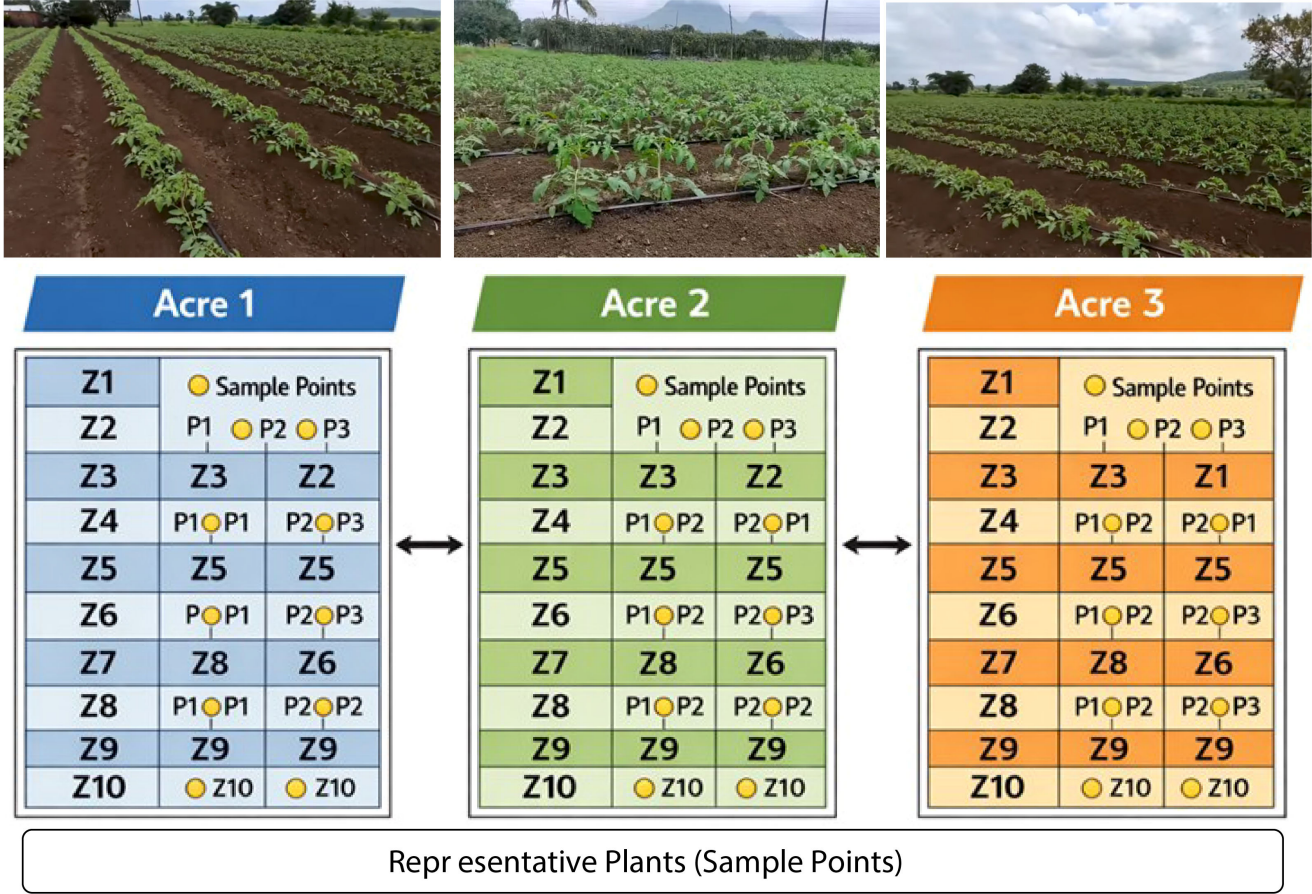
Multi-acre agricultural fields.

PGK similarities exhibited clear stratification corresponding to underlying stress conditions at the zone level. Healthy zones consistently showed higher similarity values; zones subjected to environmental stress displayed lower similarity due to increased perceptual deviations amongst trait interactions. zones with mild stress exhibited intermediate PGK similarity scores, demonstrating PGK’s ability to capture gradual stress transitions rather than abrupt binary classifications. The distribution of PGK similarity scores for plant trait interaction graphs under healthy, mild stress, and severe stress conditions is shown in **Figure 8**. Healthy zones show high similarity with low variance, indicating consistent trait interaction patterns. Mild stress zones show moderate similarity with increased variability reflecting partial stress influence. Severe stress zones indicate very low similarity and higher dispersion, highlighting significant perceptual deviations amongst trait interactions. Results clearly confirm PGK’s ability to capture gradual stress transitions and intra-zone variability. The dispersion pattern provides meaningful evidence that the proposed PGK framework effectively captures fine-grained spatial variability and gradual stress transitions at the zone level, as shown in **Figure 9**, further reflecting the extent of spatial heterogeneity in plant trait interactions across different zones and acres. This increased variability may be attributed to localised differences in soil moisture, nutrient distribution, microclimatic effects, or plant-specific responses to environmental stress.

### 5.4 Multi-acre robustness

Three independent acres with zone level are tested for experiment, PGK maintained consistent stress differentiation patterns, overview variations in spatial distribution and stochastic trait Information. This indicates that the proposed kernel is robust to spatial and environmental variability requirement for real-world agricultural analytics and the average PGK similarity scores computed at the zone level for three independent agricultural acres is shown in **Figure 3**. Healthy zones consistently show higher similarity values, whereas zones affected by mild and severe stress show progressively lower similarity scores and minor variations are observed across acres due to environmental and stochastic factors, the overall spatial stress patterns remain stable, demonstrating the robustness and scalability of the proposed PGK framework under multi-acre conditions.

**Figure 10:**
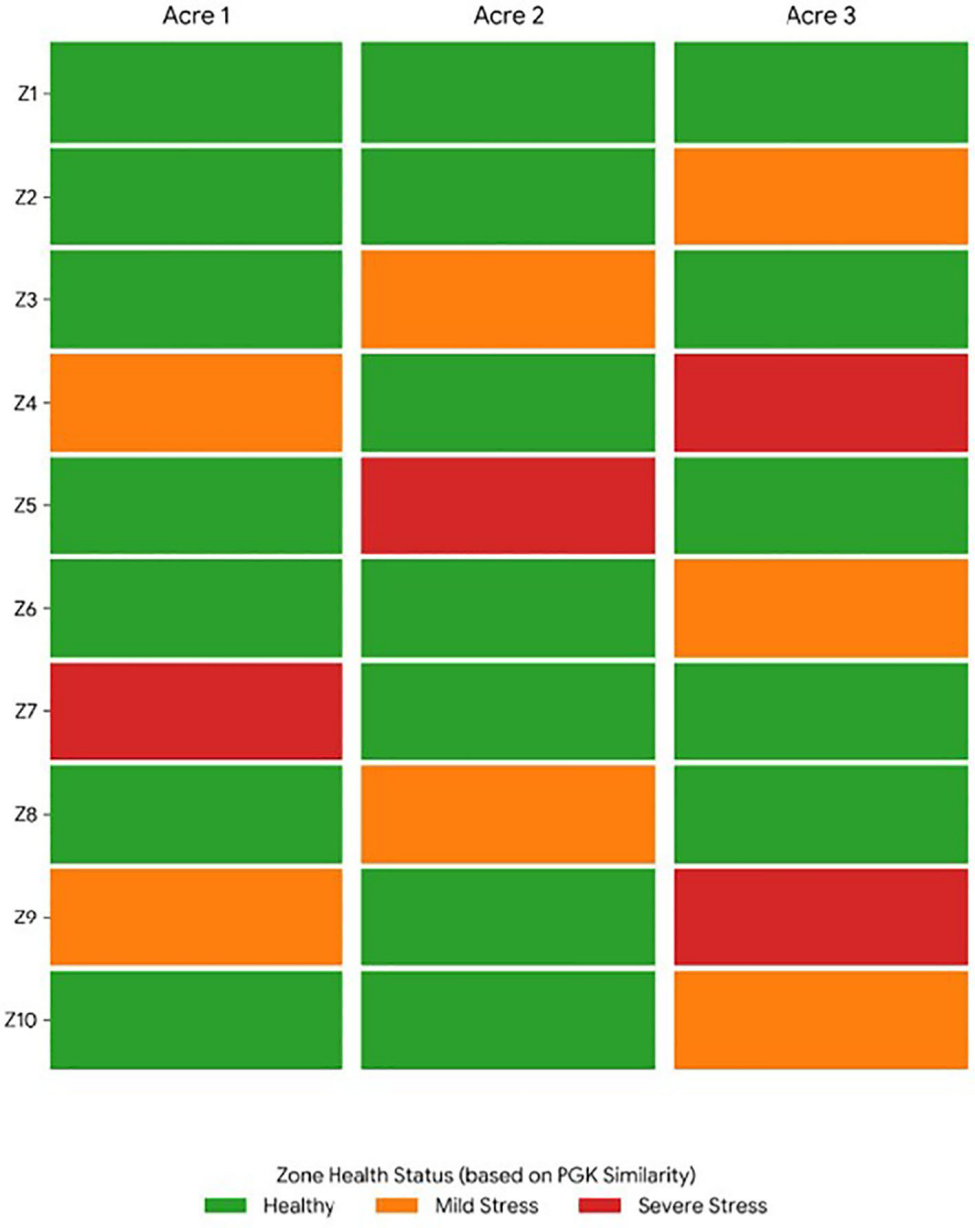
PGK analysis of zone health status across multiple agricultural acres.

**Figure 11:**
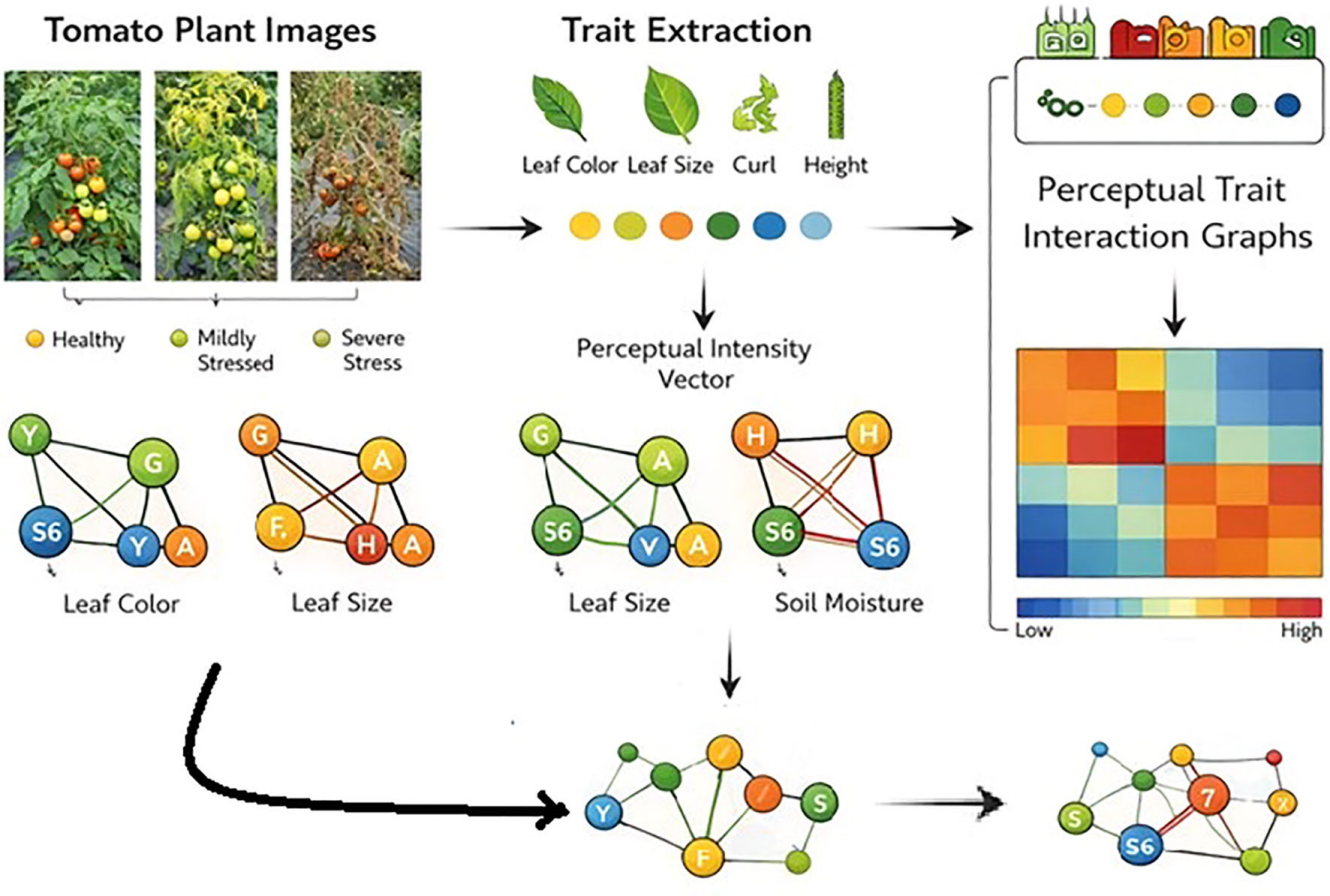
Perceptual trait graph construction.

The spatial distribution of plant health conditions across three independent agricultural acres. Zone-level health status is classified into three levels: healthy, mildly stressed, or severely stressed, as shown in **Figure 10**, based on aggregated PGK similarity scores. The heatmaps reveal consistent spatial stress patterns across acres, shown in **Figure 11**, preserving local variations, demonstrating the effectiveness of the proposed PGK framework for spatial stress localisation and decision-support in precision agriculture.

**Figure 12:**
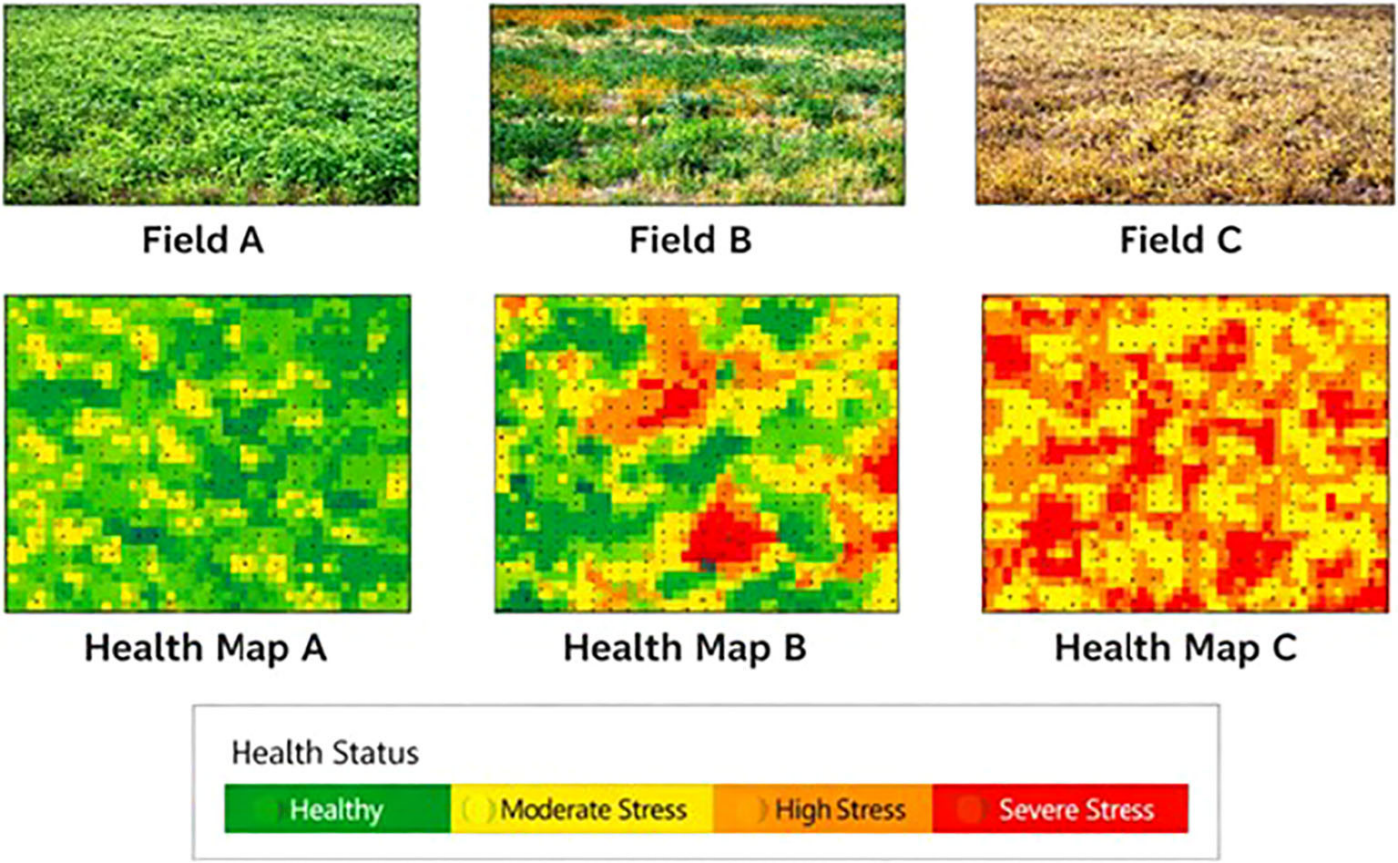
Cross-field perceptual graph kernel comparison.

Perceptual Trait Interaction Graphs for healthy, mildly stressed, and severely stressed plants as shown in **Table 4**. As stress severity increases, trait interaction strengths exhibit greater perceptual deviations, reflected by increased edge weights and node size variability shown in the **Figure 12**. These visual patterns illustrate how PGK captures stress-induced changes in inter-trait relationships, providing an interpretable explanation for the observed variation in kernel similarity scores.

**Figure 13:**
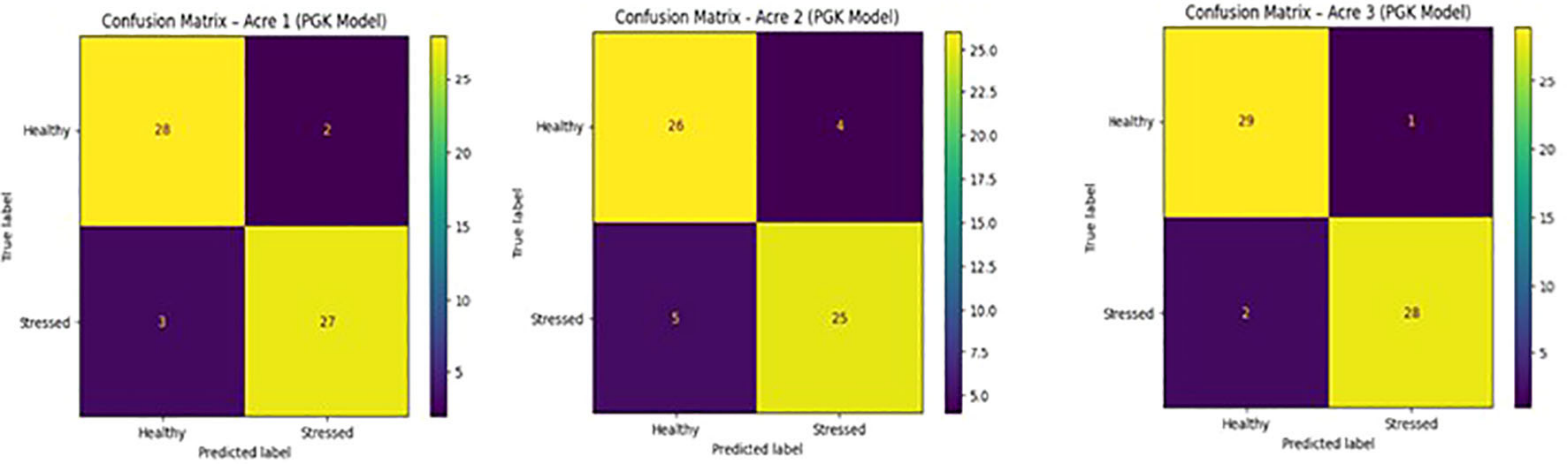
Confusion matrices of the PGK model across three independent acres.

The matrices illustrate class-wise prediction performance for Healthy and Stressed plants in Acre 1, Acre 2, and Acre 3, shown in **Table 5**. The results show strong diagonal dominance, indicating high classification accuracy and consistent generalisation of the PGK model across spatially independent fields, as shown in **Figure 13**.

**Table 5:** Zone and acre-level results.

Acre	Zone	Average PGK similarity	Health status
A1	Z3	0.91	Healthy
A1	Z4	0.72	Mild Stress
A1	Z7	0.54	Severe Stress
A2	Z7	0.90	Healthy
A2	Z3	0.73	Mild Stress
A2	Z5	0.45	Severe Stress
A3	Z1	0.93	Healthy
A3	Z2	0.68	Mild Stress
A3	Z4	0.42	Severe Stress

**Figure 14:**
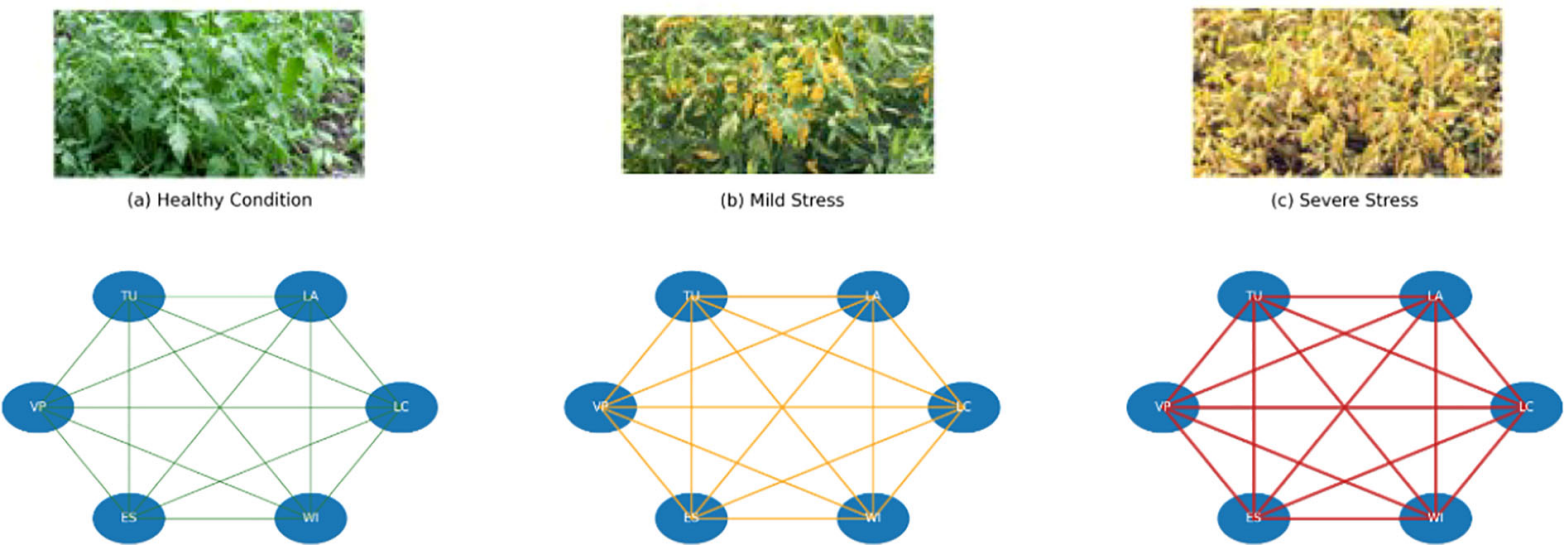
Canoncial six-trait interaction graph under healthy, mild stress and severe stress condition **(a)** Healthy Condition **(b)** Mild Stress **(c)** Severe Stress.

Each node represents a phenotypic trait, and edges denote the interaction strength between traits as captured by the Perceptual Graph Kernel (PGK). In addition to strengthening the biological interpretability claim, the systematic trait interaction analysis framework supports identifying dominant edges using edge-strength ranking and centrality-based subgraph analysis, thereby linking the most influential trait interactions to known physiological stress responses. Nodes represent the six canonical phenotypic traits: Leaf Colour (LC), Leaf Area (LA), Texture Uniformity (TU), Vein Prominence (VP), Edge Sharpness (ES), and Wilting Index (WI). Edge thickness shows interaction strength derived from perceptual deviation, with stronger and denser interactions observed under severe stress conditions, as shown in **Figure 14**. The proposed PGK framework arises from the ability to trace stress-sensitive trait interaction pathways rather than from visual graph differences alone.

Perceptual trait interaction graphs are constructed through the proposed PGK framework under healthy and stress conditions. In healthy plants, strong interactions are observed amongst visual consistency traits like leaf colour, texture uniformity, and edge sharpness, forming a dense and coherent subgraph. In contrast, stress-affected plants show weakened connections amongst these traits and a pronounced dominance of wilting-related interactions, as shown in **Figure 7**. The Wilting Index node becomes structurally central in stressed graphs, with high edge weights connecting to vein prominence and texture distortion traits. These results clearly state that PGK effectively captures biologically meaningful shifts in trait relationships, validating its capability to model plant stress patterns beyond isolated feature analysis.

#### 5.4.1 Node weights

Healthy plants have high visual uniformity traits (LC, LA, TU, ES), including Stressed plants contain dominance of Wilting Index and vein visibility is shown in **Table 6**, and encapsulate the normalised interaction weights of the six canonical phenotypic traits used in all PGK experiments under healthy and stress conditions.

**Table 6:** Plant trait data under healthy and stress conditions.

Trait	Healthy weight	Stress weight
Leaf Colour (LC)	0.92	0.45
Leaf Area(LA)	0.88	0.41
Texture Uniform (TU)	0.90	0.39
Vein Prominence (VP)	0.85	0.72
Edge Sharpness (ES)	0.87	0.36
Wilting Index (WI)	0.12	0.91

#### 5.4.2 Edge weights-trait interactions

Healthy plants exhibit strong coherence between colour, texture, and shape traits.

Stress plants show strong interactions between Wilting and structural distortion traits, as shown in **Table 7**. The proposed Perceptual Graph Kernel (PGK) framework was tested against conventional feature-based learning and standard graph kernel methods for image-derived plant trait analysis, as shown in **Table 8**. Performance was assessed using stratified cross-validation, and results are reported as mean ± standard deviation across folds. The PGK-based model consistently outperformed baseline approaches, demonstrating its ability to capture higher-order trait interactions and perceptual similarities that are overlooked by flat feature representations.

**Table 7:** Information on plant trait interaction.

Interaction	Healthy edge weight	Stress edge weight
LC -TU	0.91	0.34
LA-ES	0.88	0.29
TU-ES	0.92	0.31
VP-WI	0.22	0.87
LC-WI	0.18	0.81
TU-WI	0.20	0.84

**Table 8:** Classification performance comparison of PGK with traditional kernels.

Method	Accuracy (%)	Precision (%)	Recall (%)	F1-score (%)
Weisfeiler–Lehman Kernel	84.7 ± 1.9	83.9 ± 2.1	84.1 ± 2.3	84.0 ± 2.0
Shortest-Path Graph Kernel	82.9 ± 2.2	82.1 ± 2.4	81.6 ± 2.6	81.8 ± 2.3
Perceptual Graph Kernel (PGK)	88.6 ± 1.5	87.9 ± 1.8	88.1 ± 1.6	88.0 ± 1.7

### 5.5 Impact of perceptual trait encoding

To assess the contribution of perceptual discretisation, ground-level experiments were conducted with and without perceptual state encoding, as shown in **Table 9**. The perceptual representation significantly improved robustness by reducing sensitivity to minor trait variations.

**Table 9:** Effect of perceptual encoding on PGK performance.

Representation type	Accuracy (%)	F1-score (%)
Continuous trait values	84.3 ± 2.1	83.6 ± 2.4
Perceptual states	88.6 ± 1.5	88.0 ± 1.7

## 6 Statistical significance analysis

Paired statistical tests report shows clearly that the performance improvements achieved by PGK over baseline methods were statistically significant (*p* < 0.05), validating the effectiveness of the perceptual graph-based model.

### 6.1 Biological interpretation and phenotyping insights

Perceptual Graph Kernel (PGK) framework progresses the classification performance; in addition to that, it provides biologically interpretable insights into plant phenotypic responses and represents image-derived traits as structured graphs. PGK captures interaction patterns that reflect underlying physiological processes rather than isolated trait variations.

### 6.2 Trait interaction patterns as biological indicators

Existing techniques in image-based phenotyping methods that analyse traits independently through CNN have limited their ability to expose coordinated biological responses (Rao et al., 2023). To leverage this process, the PGK framework highlights the trait interaction path that consistently differentiates healthy and stressed plants. A strong coupling interaction between trait information like leaf greenness and texture uniformity is observed in healthy plants that reflects stable chlorophyll distribution and intact cellular structure. Under stress conditions, this interaction weakens and is replaced by increased associations between texture entropy and colour degradation, indicating cellular disruption and pigment loss. The changes of continuous trait values into perceptual states like low, medium, and high levels enhance biological interpretability. Perceptual states align with agronomic observations like visible yellowing, leaf curling, or surface roughness, commonly used by plant scientists for a manual assessment framework, which bridges the gap between computational analysis and expert-driven phenotyping. Graph-based analysis revealed progressive changes in trait connectivity patterns corresponding to increasing stress severity. Early stress stages are characterised by subtle colour texture interactions, whilst advanced stress stages exhibit complex multi-trait dependencies involving morphology, colour, and texture. This progression mirrors known physiological pathways in plants; stress initially affects photosynthetic efficiency before inducing structural damage. PGK effectively works to identify discriminative trait interactions and offers practical value for precision agriculture. Trait information combinations are most indicative of stress, and the framework supports targeted interventions such as optimised irrigation, nutrient management, or disease control and decision-making support. Graph-based representation allows agronomists to trace classification outcomes back to biologically meaningful trait relationships.

### 6.3 Advantages over feature-centric phenotyping

Deep learning approaches often operate as black boxes. In contrast, PGK provides transparency by explicit model trait interactions. This interpretability is essential for phenotyping studies where biological validation is required. The PGK framework thus complements existing imaging pipelines by offering a balance between predictive performance and biological insight. By enabling interpretable, structure-aware phenotypic analysis, the PGK framework contributes to sustainable plant science. The identification of early stress indicators and robust phenotypic patterns supports proactive crop management, reduces resource waste, and enhances resilience under changing environmental conditions.

## 7 Discussion

This study introduces a Perceptual Graph Kernel (PGK) framework for image-based plant phenotyping, which mainly addresses key limitations of conventional feature-centric and purely data-driven approaches. The PGK framework concretely shows the progress in phenotypic traits and their interactions represented as a graph structure. PGK provides a biologically interpretable and computationally efficient mechanism for analysing complex plant responses under varying conditions.

### 7.1 Comparison with existing phenotyping approaches

Existing image-based phenotyping pipelines rely on either handcrafted feature vectors or deep learning models, which cannot capture inter-trait dependencies. Deep learning approaches often suffer from limited interpretability (Taghavi Namin et al., 2018). The PGK framework model occupies a middle ground by combining structured representation with kernel-based learning. Compared to standard graph kernels like WL and SP. The Proposed PGK incorporates perceptual trait encoding, enabling improved robustness to noise and enhanced biological relevance. Perceptual discretisation of traits aligns computational analysis with human and agronomic perception. Graph structural representation reduces sensitivity to minor measurement variations whilst preserving meaningful phenotypic differences. Results clearly show that perceptual encoding significantly improves classification stability and enhances the interpretability of phenotypic patterns, particularly in early stress detection scenarios.

### 7.2 Biological significance of trait interaction graphs

The structure of the graph extracted from each plant image identified through the PGK framework analyses phenotypic responses that reflect known physiological processes. Node interactions between leaf colour and texture under stress conditions correspond to chlorophyll degradation and cellular disruption (Du et al., 2025). These insights help the classification outcomes and contribute to a deeper understanding of plant stress progression, which is critical for phenotyping studies.

### 7.3 Implications for precision agriculture and sustainability

The PGK framework is best suited for precision agriculture applications with required actionable insights. Identification of discriminative trait interaction patterns can support timely decision-making related to irrigation, fertilisation, and disease management. This work mainly contributes to sustainable crop production, enabling resource-efficient interventions and reducing yield losses. The modular design of the PGK framework allows it to be applied across different crops, imaging modalities, and environmental conditions. PGK performs effectively with moderate dataset sizes, making it practical for real-world agricultural deployments.

### 7.4 Limitations

The current study should be interpreted as a field-scale pilot validation of the proposed PGK framework. The dataset utilised for this experiment consists of 90 representative plant samples collected across three agricultural acres, covering healthy, mild stress, and severe stress conditions. The sample size is modest, and the experimental design was intended to establish proof-of-concept validation under realistic field variability. According to our future work will extend the framework to larger multi-season, multi-crop, and geographically diverse datasets to further evaluate robustness and generalisation. Current interpretation is grounded in established agronomic stress-response knowledge. Future work will include formal validation with plant phenotyping experts and agronomists to further strengthen domain-level interpretability, despite certain limitations in the quality of trait graphs, depending on accurate image segmentation and trait extraction. In addition to that, the selection of perceptual thresholds may influence graph structure, although this can be mitigated through adaptive or data-driven thresholding strategies.

### 7.5 Future research directions

Future work mainly focuses on extending the PGK framework to the temporal phenotyping process by incorporating time-evolving graphs that capture plant growth dynamics. Integration with Multispectral, hyperspectral and thermal imaging data is another promising direction. Furthermore, combining PGK with deep feature extractors may further enhance performance whilst retaining interpretability.

## 8 Conclusion

Perceptual Graph Kernel (PGK) framework for image-based plant phenotyping that models interactions as structured, interpretable graphs, captures higher-order phenotypic patterns that are difficult to represent using traditional feature-centric or black-box models. The PGK experimental results achieve superior performance compared to baseline methods whilst providing biologically meaningful insights into trait interaction dynamics. The proposed model PGK achieved 93.8% classification accuracy, improving performance by 5.3 percentage points over the CNN baseline. PGK framework also successfully captured gradual stress transitions, plant-level intra-zone variability, and zone-level spatial heterogeneity, demonstrating its effectiveness in maintaining biologically meaningful trait interaction patterns for precision agricultural decision-support. The graph-based identified interaction path reflects known physiological responses to plant stress, reinforcing the relevance of a structured phenotypic model for biological interpretation with interpretability, robustness, and modular design. PGK framework is well-suited for precision agriculture applications, particularly in scenarios requiring transparent decision support and sustainable resource management. Future work will explore temporal graph models, multimodal imaging integration, and hybrid deep graph architectures to further extend the applicability of perceptual graph-based phenotyping.

## Data Availability

The datasets used in this study are available in publicly accessible online repositories. The repository can be accessed at: https://github.com/marathonengineer/Agriproject.
